# Knowledge and Attitudes of Postpartum Mothers towards Immunization of Their Children in a Lithuanian Tertiary Teaching Hospital

**DOI:** 10.3390/medicina54010002

**Published:** 2018-03-12

**Authors:** Milda Šeškutė, Eglė Tamulevičienė, Giedra Levinienė

**Affiliations:** Department of Paediatrics, Medical Academy, Lithuanian University of Health Sciences, Eivenių str. 2, LT-50009 Kaunas, Lithuania; egle.tamuleviciene@gmail.com (E.T.); giedralev@gmail.com (G.L.)

**Keywords:** vaccines, children’s immunization, vaccine-preventable diseases

## Abstract

*Background:* Sufficient vaccination coverage among children depends on parents’ knowledge and attitudes towards immunization and their intention to have their children vaccinated. The objective of the study was to evaluate postpartum mothers’ knowledge and attitudes towards children’s immunization. *Methods:* It was a cross-sectional survey. The anonymous questionnaire was handed out to postpartum mothers selected at random in the Hospital of Lithuanian University of Health Sciences Kauno Klinikos from March to July of 2014. In total, 300 women were surveyed. *Results:* The majority (63%) of respondents had higher education. The child was the first one for 49.7% of the mothers. The women indicated that their main sources of information about children’s vaccination were the doctor, the Internet and mass media. Most respondents (87.3%) considered vaccine-preventable diseases to be dangerous but only 57.3% of them knew that vaccines provided efficient protection. Only 57% of the respondents considered vaccines to be safe but 75.3% thought that the benefits of vaccines were greater than the risks. We evaluated the knowledge as good in 36.3%, average in 41.3% and poor in 22.3% of mothers. Most of the respondents (81.3%) planned to immunize their child in the future with all the vaccines included in the national immunization program, however, 72.7% were worried about possible adverse events following vaccination. Of the mothers whose knowledge was evaluated as good, 74.8% had never refused or had doubts about having their child immunized (τ = −0.198, *p* < 0.001). The mothers with better knowledge were also less likely to be concerned (τ = 0.211, *p* < 0.001). *Conclusions:* Evaluation of postpartum mothers’ knowledge and attitudes towards children’s immunization could be the tool for better communication between health professionals and parents leading to increased vaccination rates.

## 1. Introduction

Immunization remains one of the most important public health interventions and a cost-effective strategy to reduce both the morbidity and the mortality associated with infectious diseases [1]. Decades of successful immunization programs have made vaccine-preventable diseases rarer and reduced the importance of their consequences. As a result, attention to vaccines safety has significantly increased. Sceptics’ claims about possible immunization harm continue to appear in the media even though vaccine development, production, supply control and the vaccine adverse event reporting system guarantee the quality and the safety of the vaccines [2]. Diminished direct experience of once-common childhood illnesses and increased safety concerns have caused mistrust in vaccines and have become the reasons for insufficient immunization coverage.

Vaccination coverage in Lithuania according to the national immunization program was 94–99% in various age groups until 2009. Through the following 3 years growing hesitancy and decreasing vaccination rates were observed. Fortunately, after 2012 the coverage seems to be rising again but the target to increase the scale of immunization up to 97–98% has not yet been reached [3,4]. The tendencies of decreasing immunization rates lead to the decision in 2014 for mandatory vaccination of children before attendance at day care centres in Lithuania. That again provoked discussions between anti-vaccine activists and health care specialists. Unfortunately, mandatory immunization never came into force as the Supreme Administrative Court of Lithuania determined that according to the Constitution of Lithuania parents should be free to decide whether to vaccinate the child. Vaccine-preventable diseases and insufficient immunization of children also remain an issue in other countries [5,6].

In order to identify factors which may lead to a drop in vaccination rates it is important to periodically evaluate parental knowledge and attitudes towards immunization of children [7]. Nevertheless, little data is available on the topic in Lithuania. To our knowledge, a similar study evaluating knowledge and attitudes of parents of pre-school and school-aged children was done only a decade ago [8]. As the immunization of the child starts immediately after birth (first 2 vaccines of hepatitis B and BCG are administered during the first 72 h of life), the attitudes of mothers become particularly relevant during this decision-making period. So, we aimed to evaluate current postpartum mothers’ knowledge and attitudes towards children’s immunization.

## 2. Materials and Methods

It was a cross-sectional survey that was held in the Department of Obstetrics and Gynaecology of the Hospital of Lithuanian University of Health Sciences Kauno Klinikos from March to July of 2014. It is one of the biggest centres where women are admitted from all regions of Lithuania for physiologic and pathologic deliveries (around 3000 births/year). To perform the study, we received approval (No BEC-MF-334) from the Bioethics Centre of Lithuanian University of Health Sciences.

We prepared a questionnaire that consisted of 4 questions about demographic indicators (age, education, number of children, place of residence), 20 multiple-choice questions about the knowledge and attitudes of mothers towards immunization of children (sources of information, trust in doctor, intentions to vaccinate, vaccines included in the national immunization program, optional vaccines, danger of vaccine-preventable diseases, vaccine effectiveness, safety, benefit-risk ratio, adverse events etc. (Table 1)) and one open-ended question about the reasons why mothers decided to accept or refuse vaccination of their children.

The questionnaire was pre-tested with 30 postpartum mothers in order to correct unclear questions. Then the anonymous questionnaire was handed out to the respondents selected at random among mothers hospitalized in selected postpartum wards after the delivery with their healthy newborns, during scheduled visits 2–3 times a week. The completed questionnaires were collected during the following visits. In total 300 women were surveyed in 4 months (response rate 85%).

When analysing the results, the population was divided into two groups: mothers bearing the first child, including twins (primiparous) and mothers bearing the second or more children. Maternal level of education for analysis was also divided into two major groups: (1) unfinished secondary and secondary and (2) post-secondary. We evaluated mothers’ knowledge by summing their correct answers (1 point per correct answer) to 6 questions: vaccines included in the national immunization program, optional vaccines, danger of vaccine-preventable diseases, vaccine effectiveness, safety and benefit-risk ratio. Mothers’ knowledge was evaluated as poor (scoring 0–2 points), average (3–4 points) or good (5–6 points).

Statistical analysis was performed using SPSS software version 20.0. We used a chi-square test to determine dependence of qualitative variables and Kendall’s coefficient to evaluate correlation. We defined statistical significance as *p* < 0.05.

## 3. Results

### 3.1. Demographic Characteristics

We surveyed 300 mothers who were 15 to 49 years old (the mean age—29.51 ± 5.589 years). The majority (63%) of them had higher education and lived in the urban areas (77.7%). Half of the mothers were primiparous (49.7%) (Table 2).

### 3.2. Evaluation of Mothers’ Knowledge

**Figure 1:**
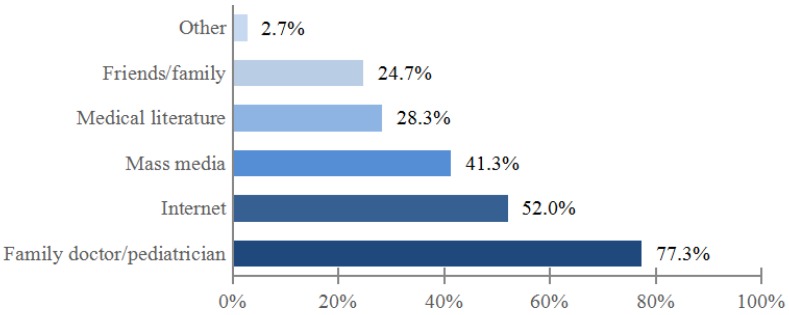
The main sources of information on children’s immunization indicated by the mothers (few answers were possible).

**Figure 2:**
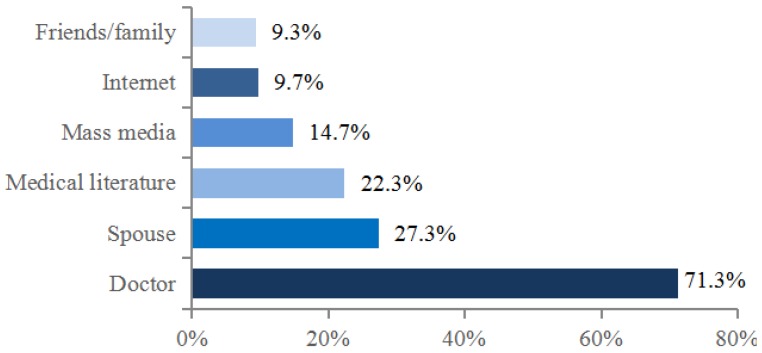
Sources indicated by the mothers that had the biggest impact on their decision to vaccinate their children (few answers were possible).

Doctor was indicated as the main source of information about children’s vaccination by majority (77.3%) of mothers (Figure 1). The physician also had the biggest influence on mothers’ decisions to have their child vaccinated. Other persons or sources were indicated much less often (Figure 2).

More than half (62.1%) of the respondents answered that the family doctor/paediatrician provided all the information they needed about child’s immunization, whereas 13.8% thought that the information was not sufficient. Majority (74.5%) of the mothers wanted more information.

We found that 73.1% of the respondents trusted the information regarding vaccination of children provided by the family doctor/paediatrician, while 19.5% doubted—mostly primiparous mothers (70.7%). Among mothers who did not trust the information on vaccination given by the doctor (7.4%), a majority (68.2%) had more than one child.

Maternal knowledge about the vaccines included in the national immunization program and about optional vaccines was similar. Most respondents (87.3%) considered vaccine-preventable diseases to be dangerous but only 57.3% of them considered the vaccines to effectively protect the child from these diseases. A similar number of mothers (57%) knew vaccines were safe (Table 3). However, 75.3% of the survey participants answered that benefits of immunization outweighed the risks.

Maternal level of education was directly associated with their knowledge about optional vaccines (χ² = 18.860, df = 3, *p* < 0.001; τ = 0.226, *p* < 0.001), disease dangerousness (χ² = 27.257, df = 6, *p* < 0.001; τ = 0.269, *p* < 0.001) and benefit-risk ratio of immunization (χ² = 15.195, df = 6, *p* = 0.019; τ = 0.162, *p* = 0.003) but had no significant relationship with mother’s knowledge on the vaccines included in the national immunization program, vaccine safety or effectiveness. We did not find any significant correlations between mother’s age, number of children or place of residence and mother’s answers to these 6 separate questions.

Eventually, we evaluated maternal knowledge by summing up correct answers to the aforementioned questions: 36.3% of mothers were evaluated as having good knowledge, 41.3% as average and 22.3% as poor. The associations between mothers’ knowledge and their age, place of residence or the number of children again did not reach statistical significance. The only significant relationship was observed between maternal knowledge of children’s immunization and the mother’s education (χ² = 21.415, df = 6, *p* = 0.002): the mothers with higher level of education had better knowledge (τ = 0.193, *p* < 0.001).

### 3.3. Evaluation of Mothers’ Attitude

Most respondents (85.3%) were worried about their child’s immunization. When asked what makes them most concerned, 72.7% of mothers indicated possible adverse reactions of vaccines (mostly influenza, hepatitis B, BCG and MMR). Less respondents were worried that the vaccine could cause the disease that the child was immunized against (15.3%), that a few vaccines would be injected at the same time (15.3%) or that immunization could be painful for the child (11.7%). Though adverse events were the main concern of mothers, 77% of respondents answered that they had never known anyone who had experienced any adverse events following immunization nor had their children experienced it. Only 4.4% of mothers reported that their children had had adverse events and 18.6% knew someone who had experienced it. Surprisingly, we did not find any association between mothers’ experience of adverse events and their concerns about children’s immunization. On the other hand, maternal knowledge and their concerns about immunization were associated (χ² = 14.963, df = 2, *p* = 0.001): respondents with better knowledge were less likely to be concerned (τ = 0.211, *p* < 0.001).

**Figure 3:**
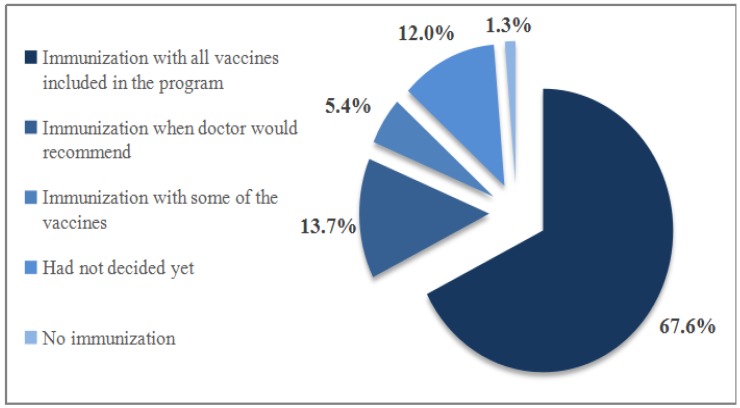
Mothers’ intentions to vaccinate their child with the vaccines included in the national immunization program.

The majority of mothers (83.2%) reported that their overall opinion of children’s immunization was positive (4% were negative, 12.8% had no opinion). Mothers’ intentions to vaccinate their child with the vaccines included in the national immunization program are shown in Figure 3.

One open question was given to the mothers to analyse the reasons of the decision to accept or refuse their child’s vaccination. Two thirds (68.4%) of the respondents answered the question, mostly mothers, who agreed to immunize their child. The most common reasons to vaccinate were protection of the child (51.3%), perceived severity of illness (22.8%) and belief in benefits of the vaccine (21.2%). Only 4% of respondents indicated reasons for refusal of immunization: perceived ineffectiveness of vaccines and safety concerns.

We found a few factors related to mothers’ decision of immunization. Study analysis showed an association between mothers’ knowledge and their decision to have their child vaccinated (χ² = 15.729, df = 4, *p* = 0.003): 74.8% of mothers, whose knowledge was evaluated as good, had never refused or had doubts about having their child immunized (τ = −0.198, *p* < 0.001). There were also significant direct associations between vaccine refusal and perceived vaccine safety (χ² = 135.081, df = 4, *p* < 0.001) and efficacy (χ² = 92.697, df = 4, *p* < 0.001): those mothers who answered that vaccines were unsafe or ineffective, significantly more often refused to vaccinate their child (τ = 0.391, *p* < 0.001; τ = 0.367, *p* < 0.001). Trust in doctor-given information was associated with the decision to have the child vaccinated (χ² = 29.014, df = 2, *p* < 0.001): the decision of refusing vaccination has been significantly less common among the mothers who trusted information given by the doctor than among those who had not (τ = −0.151, *p* = 0.008).

## 4. Discussion

The strength of our study was to analyse the knowledge and attitudes of mothers towards immunization of children during the early phase of the decision-making process. The study revealed that mothers’ lack of correct information is a significant cause of doubts and negatively influences their decision regarding immunization of the child. Unfortunately, even in a country with high vaccine coverage, most of the mothers have concerns regarding immunization and vaccine safety. Nevertheless, according to the survey participants, the doctor still plays the most important role in their choice.

The most popular source of information about children’s immunization according to majority of respondents (77.3%) was the doctor. This is in line with surveys done in other countries [10,11,12,13,14]. For example, the doctor was chosen as the main source of information by 81.7% of respondents in the US (together with the nurse) [12] and by about 91% of survey participants in Austria [13].

We also evaluated what made the biggest impact on the decision to immunize the child. Though nearly half of the respondents indicated mass media or the Internet as one of the main sources of information, our study revealed that it rarely played the most important role on the decision (14.7% and 9.7% respectively). Compared to other sources, the doctor was the most influential (71.3%). According to the review of literature containing European data, a health care professional’s advice was the most commonly cited reason for general population support for vaccination [10]. It confirms that the doctor’s role is pivotal in both giving the right information and helping parents to make an appropriate decision.

Our study focused on the views of mothers because traditionally they are the key decision makers on the issue [13,15,16]. The spouse’s opinion determined the mother’s decision only in one third of the cases, though new studies done in New Zealand and Japan showed that fathers could help to improve vaccination coverage [17,18]. The reason that fathers’ input into decision making is lower could be that, compared to mothers, they have fewer opportunities to interact with health care specialists [17]. Thus, measures should be aimed at delivering knowledge to both parents.

The study revealed that most of the mothers lack sufficient information about immunization—only around a third of them were well informed. Limited maternal knowledge was also observed in other studies [8,15,19], however, correct knowledge is crucial in making the right decision whether to have the child vaccinated.

Good knowledge was one of the most important factors that determined positive attitudes towards vaccination in our study. Mothers who had better knowledge were less likely to refuse or doubt their child’s immunization. Though our data analysis showed that better knowledge was influenced by higher education, according to the findings of recent studies carried out in other developed countries, higher education is associated with negative attitudes towards vaccination [13,20]. The systematic review of Yaqub et al. explains this phenomenon by well-educated parents in developed countries harbouring more hesitant attitudes because of increased distrust in health care providers [10]. However, it seems that so far in our country lack of knowledge or misinformation plays the most important role.

As already mentioned, not only the knowledge of the mother but also the trust in the doctor (the main source of information) could influence a mother’s decision to have the child vaccinated: 73.1% of our respondents indicated that they trust the information given to them by the family doctor or paediatrician. During a Lithuanian citizens’ survey in 2011 it was found that 79% of women of various ages trusted the information on immunization given by the physician [21]. Similar results were obtained in a study that was carried out in the USA: 76% of surveyed parents trusted information given by the child’s doctor [22]. Meanwhile during the VACSATC (Vaccine Safety, Attitudes, Training and Communication Project) study that was performed in a few European countries it was found that trust in physicians ranged from 54% in Norway to 92% in England [7]. These differences could have been influenced by gender, because women are likely to trust doctors more and in Norway men comprised a larger part of the respondents. Also, study methods may have had an influence: parents in England were interviewed face-to-face while in Norway questionnaires were sent by mail. The Norwegians’ attitude results also could have been biased by the low response rate—only 40%.

Since vaccines are administered to healthy children (not to treat but to prevent currently rare diseases), safety of the vaccines seems to be one of the biggest parental concerns regarding a child’s immunization: nearly 6 out of 7 respondents were worried about the child’s vaccination, the majority of them being the most concerned about the risk of adverse events. Concerns regarding immunization were more likely to be connected with misinformation rather than unpleasant direct experience: 57.0% of our surveyed mothers knew that vaccines administered to their children were safe while their experience of vaccine-induced adverse reactions and their concerns about child’s vaccination were not significantly related. A similar study done in Lithuania nearly a decade ago showed a better opinion about the safety of vaccines [8].

Although mothers’ opinions about the immunization of children are generally positive, even among those mothers who had their children vaccinated, nearly one of three were uncertain about their decision. The number of hesitant parents is increasing all over Europe: more than one in five of parents in England, Poland and Sweden reported that they had had doubts about vaccinating their children [7]. It shows that even the mothers who agree to have their child vaccinated can have many doubts regarding certain aspects of immunization. The concern is that growing hesitancy can lead to increased refusal. To maintain sufficient vaccination coverage in the future it is necessary to pay special attention to the reasons for hesitancy of immunization and to take measures to reduce the percentage of doubters.

We hope that the results of our survey will help health professionals to communicate better with parents about immunization and provide them with information based on scientific evidence and official recommendations.

Our study has some limitations. First, even though our surveyed mothers represent well the Lithuanian mothers’ population according to the age at childbirth and the number of children, the study population is rather small for a cross-sectional survey. Second, the answers of surveyed postpartum mothers could have been influenced by physical and emotional factors after the delivery. On the other hand, that is the time when they actively take the decision to vaccinate their child with the first vaccines—BCG and hepatitis B. Third, our results could as well have been distorted by a relatively large number of primiparous mothers who might have not discussed vaccination of the child with their doctor before. Also distributing the survey in the hospital did not include women who give birth at home. At the same time, it should be noted that the number of such women in Lithuania is small.

## 5. Conclusions and Implications

Evaluation of postpartum mothers’ knowledge and attitudes towards children’s immunization is the tool for better communication between health professionals and parents. The doctor was the main source of information about children’s vaccination and also had the biggest influence on the mother’s decision to have their child vaccinated. So, it is important that continuous training of health-care specialists would include evidence-based information and official recommendations about children’s vaccination.

Health professionals should be encouraged to engage in better communication with parents, starting from the pregnancy period and paying special attention to less educated mothers, because one third of postpartum mothers had insufficient knowledge or were misinformed.

Although the mothers’ opinions about children’s vaccination were generally positive, a majority of them had concerns regarding vaccine safety which were not based on direct experiences. That is why parents’ trust in immunization would be improved with a more active and transparent surveillance system of vaccine adverse events, accessible to the parents.

## Figures and Tables

**Table 1:** List of the multiple-choice questions used in the questionnaire.

1. Do you search for information about immunization of children by yourself? 2. What are your main sources of information about immunization of children? 3. How would you evaluate your knowledge about immunization of children? 4. Does your family doctor/paediatrician provide all the information you wonder about child’s immunization? 5. Do you trust the doctor-given information about immunization of children? 6. Who (what) has the biggest impact on your decision to vaccinate the child? 7. Have you ever rejected or doubted whether to vaccinate your child? 8. Have you ever postponed your child’s immunization? 9. Do you plan to have your child vaccinated with all the vaccines included in the national immunization program? 10. From which diseases according to national immunization program can children be vaccinated for free in Lithuania? 11. From which diseases children can be vaccinated additionally in Lithuania? 12. Are the diseases which your child is being immunized against dangerous? 13. Do vaccines effectively protect your child from the diseases? 14. How would you evaluate the benefit-risk ratio of the vaccines? 15. Are the vaccines used for children immunization safe? 16. Have you ever faced or knew anyone who had faced adverse events of the vaccines? 17. In your opinion, which vaccine causes adverse events the most often? 18. What makes you the most concerned about child’s immunization? 19. What is your opinion about immunization of children? 20. Would you like to get more information about it?

**Table 2:** Demographic characteristics of the respondents compared to the Lithuanian general population in 2014.

	Study Participants		Lithuanian General Population *
**No. of Respondents**	300		-
**Mean Age at Childbirth, years**	29.51 ± 5.589		29.4
**Education Level:**	n	%	% **
(a) higher university	189	63	37.7
(b) higher non-university	20	6.7	4.9
(c) secondary	77	25.6	51.2
(d) unfinished secondary	14	4.7	6.2
Total:	300	100	100
**Child Born in the Family:**	n	%	%
1st born	149	49.7 ***	47.7
2nd born	97	32.4	37
3rd born etc.	54	17.9	15.3
Total:	300	100	100

* Statistics Lithuania [9]. ** education of women aged 25–64 years in Lithuania. *** 0.3% were twins.

**Table 3:** Mothers’ knowledge about children’s immunization.

Questions:	Yes	No	Did Not Know
	n (%)	n (%)	n (%)
Are the diseases which your child is being immunized against dangerous?	262 (87.3)	11 (3.7)	27 (9.0)
Do vaccines effectively protect your child from the diseases?	172 (57.3)	20 (6.7)	108 (36.0)
Are the vaccines used for children immunization safe?	171 (57)	15 (5.0)	114 (38.0)
